# Importance of Temporal Analyzes for the Exploration of the Posturographic Correlates of Emotional Processing

**DOI:** 10.3389/fnbeh.2018.00277

**Published:** 2018-11-15

**Authors:** Harold Mouras, Thierry Lelard

**Affiliations:** ^1^Laboratoire de Neurosciences Fonctionnelles et Pathologies, Centre Universitaire de Recherche en Santé, Amiens, France; ^2^Département de Psychologie, UFR SHSP, Université de Picardie Jules Verne, Amiens, France; ^3^Adaptations Physiologiques à l’Exercice et Réadaptation à l’Effort, UFR des Sciences du Sport, Université de Picardie Jules Verne, Amiens, France

**Keywords:** posturography, social neuroscience, affective neuroscience, motor correlates, embodiment

## Abstract

Over the last two decades, affective and social neurosciences converged on the study of motor correlates of emotional and motivational information processing. Posturography appeared as a good experimental method to address this question. The use of this method to explore emotional and motivation processing remains recent. Here, we summarize several important arguments showing the importance to explore the temporal aspects of these responses regarding the complexity of the link between emotional information’s parameters (such as arousal) and the corresponding neural processes.

The exponential development of modern neuroimaging techniques has allowed the investigation of neural processes and their peripheral correlates from new angles. At the interface of affective and social neurosciences, it seems quite clear that relationships between individuals firstly obey a motivational component of inter-attraction, which may be either positive or negative. This notion of inter-attraction can be considered in different functional contexts, such as inter-individual recognition, attachment or affiliation relationships. This underlines the strong entanglement between motor and emotional processes and has to do with the conception of emotion as an action disposition (Lang et al., 1993; Lang, 1994), inducing an approach-avoidance type behavioral response (we tend to approach what is acceptable to promote our survival and avoid painful experiences in order to protect us from harm; Elliot and Covington, 2001). Creating one of the most important emotional stimuli database, Lang (2005) rated emotional stimuli in terms of “approach-avoidance” behavior – indexed by the subject *vs.* target distance – induced either as “appetitive” or “defensive”. Within the framework of motivated social interactions, embodiment appears to be a central process. This process quickly occurs in the dynamic of psychological processes involved in motivated social interactions and we want to underline with this paper that embodiment’s modulation of the postural correlates of emotional information processing appears when considering the temporal dimension of the response.

Posturography is a suitable experimental method to assess postural control by determining the body’s Center-of-Pressure (COP) displacements (Gurfinkel, 1973). For this reason, it was used to assess the motor correlates of emotional processing by contrasting the effects of valence

and arousal of emotional stimuli on postural control (Horslen and Carpenter, 2011). Within the theoretical framework of affective neuroscience, the influence of emotion on decision-making processes (Damasio, 1996) through emotional biasing of action selection might demonstrate the functioning of a Pavlovian system that innately regulates specified responses to reward- or punishment-predictive stimuli (Ly et al., 2014) which is supported by results reporting, respectively, behavioral activation vs. inhibition in response to reward vs. punishment (Crockett et al., 2009; Guitart-Masip et al., 2011, 2012, 2014; Cavanagh et al., 2013). In recent studies, presentation of emotional pictures (International Affective Picture System, (IAPS); Lang, 2005) has been shown to induce approach-withdrawal behavior (Hillman et al., 2004) or freezing responses (Hillman et al., 2004; Azevedo et al., 2005; Lelard et al., 2013; Stins et al., 2015a). Most of these studies assessed posturographic correlates of emotional processing by calculating mean postural responses induced over a time period of 6 up to 50 s. Similarly, one decade ago, the use of modern neuroimaging techniques to explore the neural correlates of human male sexual arousal was still emergent and most studies considered recorded data through classical subtractive addressing the question of the more activated brain areas in response to visual sexual stimuli as compared to neutral ones. In new studies, we recorded simultaneously cerebral hemodynamic responses by functional neuroimaging and erectile responses by volumetric penile plethysmography (Moulier et al., 2006; Mouras et al., 2008). It allowed to perform cross-correlation analyzes between both responses leading to identify new areas as compared to classical subtractive analyzes because these analyzes integrated the dynamic (i.e., temporal courses as compared to mean values) aspects of both responses. Interestingly, this aspect describes exactly the same problematic that the one of the present manuscript, considering as central the temporality of the processes for the understanding of the complexity of the whole mechanism. However, in this short note, we want to summarize several important arguments to underline the importance to perform temporal analyzes to identify the complexity of posturographic correlates of emotional and motivational processing.

First, when posturographic parameters are plotted against time and not only averaged over a time period, this is often possible to catch the complexity of posturographic responses in term of temporal evolution and thus assess significant effects over conditions that are not apprehended through mean values analyzes. In a recent study (Lelard et al., 2014), a posterior displacement of the mean anteroposterior COP position was reported after 3 s in response to aversive stimuli as compared to neutral stimuli. Earlier, Hagenaars et al. (2014) reported a reduction of body sway path after 1.5 s in response to unpleasant video images as compared to pleasant ones. In another recent study (Lelard et al., 2017), we tested the effect of mental simulation of painful situations (thought to increase embodiment processes) on the posturographic correlates of painful situations processing. Whereas the comparison of the mean value over a period of presentation of 12 s did not allow to demonstrate a significant modulation of the painful situations posturographic correlates between a passive condition as compared to an active mental simulation one, results appeared clearer when plotting the data against time. Indeed, an interaction effect (instruction × stimuli × time) demonstrated that mental simulation induced posterior displacement of the mean position of the COP at different times during presentation of visual stimuli (4 s; 9–12 s). These temporal analyzes allowed to demonstrate a posterior displacement of the COP in the mental simulation condition compared to the passive observation condition.

Secondly, a certain amount of studies demonstrated the complexity of motor responses induced by emotional and motivational informations processing. Recently, Stins et al. (2015b) found that gait was initiated faster with pleasant images at onset, and faster with unpleasant images at offset. Gélat and Chapus (2015) demonstrated that reaction time in gait initiation depends on the time available for affective processing. Regarding the posturographic correlates of sexual information processing (Mouras et al., 2015), contrary to our primary hypothesis of an approach-type behavior in response to sexual visual stimuli in a sample of heterosexual healthy human males, we recorded a freezing-type response to sexually explicit stimuli. Even if temporal courses were not calculated in this study, this paradoxical response underlined the complexity of the motor response developed along the sexual arousal and broader along goal-directed behaviors. This demonstrated an early freezing response that was also recorded in animal research in different experimental situations and was interpreted as maybe linked to the recruitment of supplementary cognitive resources to allow the real initiation of more complex movements to move toward the goal or the target. This complexity and the necessity to integrate the temporal evolution of physiological parameters through emotional and motivational information processing is in accordance with recent theoretical models. In the “Glutamate Amplifies Noradrenergic Effects”, GANE) model, Mather et al. (2016) theorized how arousal amplifies selectivity in perception and memory through the initiation of local hotspots of neuronal excitation by norepinephrine and thus, made an implicit link between the temporality of the processes and the level of arousal of the emotional information that is being processed. Freezing has also been observed as a response to social fear. Recently, Qi et al. (2018) reported that the time courses of the establishment and extinction of social defeat were particularly consistent with the contrasting Basolateral Amygdala (BLA) and ventral Hippocampus (HIP) responses involved in this process. This temporal aspect is central as fear responses are most of the time produced through the competition of parallel neural circuits. For example, Fadok et al. (2017) demonstrated that the competition between active and passive fear responses was based on a competitive interaction between two defined populations of inhibitory neurons, a circuit motif allowing for rapid and flexible action selection.

If we consider that embodiment is a preliminary process to induce motivated social interactions, the exploration of the posturographic correlates of motivated social interactions has to integrate the temporal dimension of the responses and consider recent theoretical developments on the issue of “being together” and more specifically the issue of “being together in time” (Laroche et al., 2014). Here, we give arguments to support embodiment as a central process for motivated social interactions, modulating postural correlates of emotional information processing when looking at their temporal courses. When performing the temporal analyzes on a time interval of 12 s, it seems possible to distinguish an “early” component (around 4 s) as compared to a “later” component (around 9 to 12 s) of the embodiment’s modulation effect of the postural correlates.

## Author Contributions

All authors listed have made a substantial, direct and intellectual contribution to the work, and approved it for publication.

## Conflict of Interest Statement

The authors declare that the research was conducted in the absence of any commercial or financial relationships that could be construed as a potential conflict of interest.
